# Immunogenetic Epidemiology of Dementia and Parkinson’s Disease in 14 Continental European Countries: Shared Human Leukocyte Antigen (HLA) Profiles

**DOI:** 10.29245/2578-3009/2021/2.1209

**Published:** 2021-04-28

**Authors:** Lisa M. James, Apostolos P. Georgopoulos

**Affiliations:** 1 The HLA Research Group, Brain Sciences Center, Department of Veterans Affairs Health Care System, Minneapolis, MN, 55417, USA; 2 Department of Neuroscience, University of Minnesota Medical School, Minneapolis, MN 55455, USA; 3 Department of Psychiatry, University of Minnesota Medical School, Minneapolis, MN 55455, USA; 4 Department of Neurology, University of Minnesota Medical School, Minneapolis, MN 55455, USA

**Keywords:** Dementia, Parkinson’s disease, Human leukocyte antigen, Immunity, Genetics

## Abstract

Human leukocyte antigen (HLA), which is critically involved in immune response to foreign antigens and in autoimmunity, has been implicated in dementia and Parkinson’s disease. Here we report on the correlations between the population frequencies of 127 HLA Class I and II alleles and the population prevalence of dementia and Parkinson’s disease in 14 Continental Western European countries, extending previous work^1,2^. We used these correlations to construct and compare HLA profiles for each disease^3^. We found that (a) the HLA profiles of the two diseases were significantly correlated across both HLA Class I and Class II alleles, (b) negative (“protective”) HLA-disease correlations did not differ significantly for either HLA class, but (c) positive (“susceptibility”) HLA-disease correlations were significantly higher in dementia than in Parkinson’s disease for both HLA classes of alleles. These findings indicate that (a) dementia and Parkinson’s disease share immunogenetic HLA-related mechanisms, (b) HLA-related protective mechanisms (presumably against pathogens) do not differ between the two diseases, but (c) HLA-related susceptibility mechanisms (presumably underlying autoimmunity) are significantly stronger in dementia than in Parkinson’s disease.

## Introduction

Nearly 50 million people worldwide are living with dementia or Parkinson’s disease, the two most common neurodegenerative diseases, resulting in a considerable toll on affected individuals, caregivers, and society^4,5^. Despite decades of extensive investigation, the etiology of both conditions remains unknown, hampering intervention and prevention efforts; however, growing research points to the involvement of pathogens as potential causes^6–8^ and autoimmunity as pathogenic mechanisms that are triggered by, and subsequently contribute to, chronic neuroinflammation in both conditions^9–13^. In light of overlapping pathogen associations and autoimmune-related neuroinflammation in dementia and Parkinson’s disease, and the known involvement of the Human Leukocyte Antigen (HLA) in pathogen elimination and autoimmunity, we sought in the present study to evaluate the correspondence between the HLA disease profiles in these two diseases as an indicator of immunogenetic overlap.

The HLA system is involved in both pathogen elimination (as a preventive/protective factor) and autoimmunity (as a susceptibility factor). Two main classes of HLA genes code for cell-surface glycoproteins that are critically involved in facilitating cellular and humoral immune system responses to foreign antigens derived from various pathogens. With respect to pathogen elimination, HLA Class I molecules (of the A, B, C genes) are expressed on nucleated cells and present intracellular antigen peptides to CD8+ cytotoxic T cells to signal cell destruction, thus eliminating infected cells. On the other hand, HLA Class II molecules (of the DR-, DQ- and DP-genes) are expressed on professional antigen-presenting cells (e.g., macrophages, dendritic cells) and present endocytosed extracellular antigen peptides to CD4+ T cells to promote B-cell mediated antibody production against the offending pathogens and adaptive immunity for the future. HLA molecules are coded in the Major Histocompatibility Complex (MHC) in chromosome 6. MHC is the most highly polymorphic region in the human genome resulting in considerable individual and population variation in HLA composition, reflecting the long evolutionary history of exposure to and dealing with elimination of, and ultimate protection from, various pathogens^14^,^15^. With respect to autoimmunity, both HLA Class I and Class II molecules are intimately involved in autoimmune disorders^16^.

Burgeoning evidence has demonstrated HLA associations with dementia and Parkinson’s disease^1^–^3^,^9^,^17^–^21^. Using an across-countries population immunogenetic epidemiological approach^22^, we initially investigated associations between the population frequency of HLA Class II DRB1 alleles and dementia across 14 countries in Continental Western Europe^1^,^2^. This approach permits identification of an HLA profile wherein HLA alleles may presumably be characterized as protective alleles (negatively correlated with population prevalence of a disease) or susceptibility alleles (positively correlated with the population prevalence of a disease). With respect to HLA Class II DRB1 alleles, we recently found that the HLA profiles of dementia and Parkinson’s disease for a small number of HLA alleles are very similar, suggesting that similar immunogenetic mechanisms may underlie the pathogenesis of these conditions^3^. Here we extend that line of research to evaluate the correspondence in the association of the population frequency of 127 HLA Class I and Class II alleles and the population prevalence of dementia and Parkinson’s disease which will permit characterization of HLA-disease profiles. Our hypothesis is that, in part at least, these diseases are caused by pathogens which, if allowed sustained presence in the body, may inflict brain damage both directly (by killing cells and inflammation) and/or indirectly (by autoimmunity). Since the HLA system is involved both in pathogen elimination and autoimmunity, we focus on HLA association with disease prevalence as a “bird’s eye view” to gain an insight into the involvement of pathogen elimination and autoimmunity in the pathogenesis of PD and dementia. The key point is that a pathogen elimination effect would be manifested as a negative correlation between disease prevalence and allele frequency, whereas, in contrast, autoimmunity would be manifested as a positive correlation.

## Materials and Methods

### Prevalence of dementia and Parkinson’s disease

The population prevalence of dementia and Parkinson’s disease was computed for each of the following 14 countries in Continental Western Europe: Austria, Belgium, Denmark, Finland, France, Germany, Greece, Italy, Netherlands, Portugal, Norway, Spain, Sweden, and Switzerland. Specifically, the total number of people with dementia or Parkinson’s disease in each of the 14 Continental Western European countries as determined by the Global Burden of Disease study^4^,^5^ was divided by the total population of each country in 2016 (Population Reference Bureau)^23^ and expressed as a percentage. We have previously shown that life expectancy for these countries are virtually identical^1^; therefore, life expectancy was not included in the current analyses.

### HLA

The frequencies of all reported HLA alleles of classical genes of Class I (A, B, C) and Class II (DPB1, DQB1, DRB1) for each of the 14 Continental Western European countries were retrieved from the website allelefrequencies.net (Estimation of Global Allele Frequencies^24^,^25^) on October 20, 2020. There was a total of 2746 entries of alleles from the 14 Continental Western European countries, comprising 844 distinct alleles. Of those, 127 alleles occurred in 9 or more countries and were used in further analyses. The distribution of those alleles to the HLA classes and their genes is given in Table 1.

### Data analysis

HLA profiles for Parkinson’s disease and dementia were derived as described previously^3^. Briefly, the prevalences (fractions of total country population) of Parkinson’s disease and dementia were natural-log transformed^1^–^3^ (see below) and the Pearson correlation coefficient, r, between disease prevalence and the population frequency of each one of the 127 HLA alleles above calculated and Fisher z-transformed^26^ to normalize their distribution:

(1) $$r^{'}=atanh\;(r)$$

The HLA disease profile consisted of 127 values of $r^{'}$. The association between the HLA profiles of Parkinson’s disease and dementia was computed as the Pearson correlation between their HLA profiles. Since negative and positive correlations (-*r* +*r*) between allele frequency and disease prevalence can be interpreted as indicating a protective or susceptibility effect of the allele on the disease, respectively, additional analyses were carried out on the counts of signed (negative, positive) correlations using two-way tables and associated statistics (chi-square test, Fisher’s exact test, phi coefficient). Finally, the quantitative differences between Parkinson’s disease and dementia with respect to protective (negative $r^{'}$) and susceptibility (positive $r^{'}$) alleles were assessed by a paired t-test in two groups, namely one on alleles protective to both diseases, and the other on susceptibility alleles for both diseases. Statistical analyses were performed using the IBM-SPSS package (IBM SPSS Statistics for Windows, Version 26.0, 64-bit edition. Armonk, NY: IBM Corp; 2019) and Intel FORTRAN (Microsoft Visual Studio Community Version 16.8.3; Intel FORTRAN Compiler 2021).

## Results

**Figure 1.**
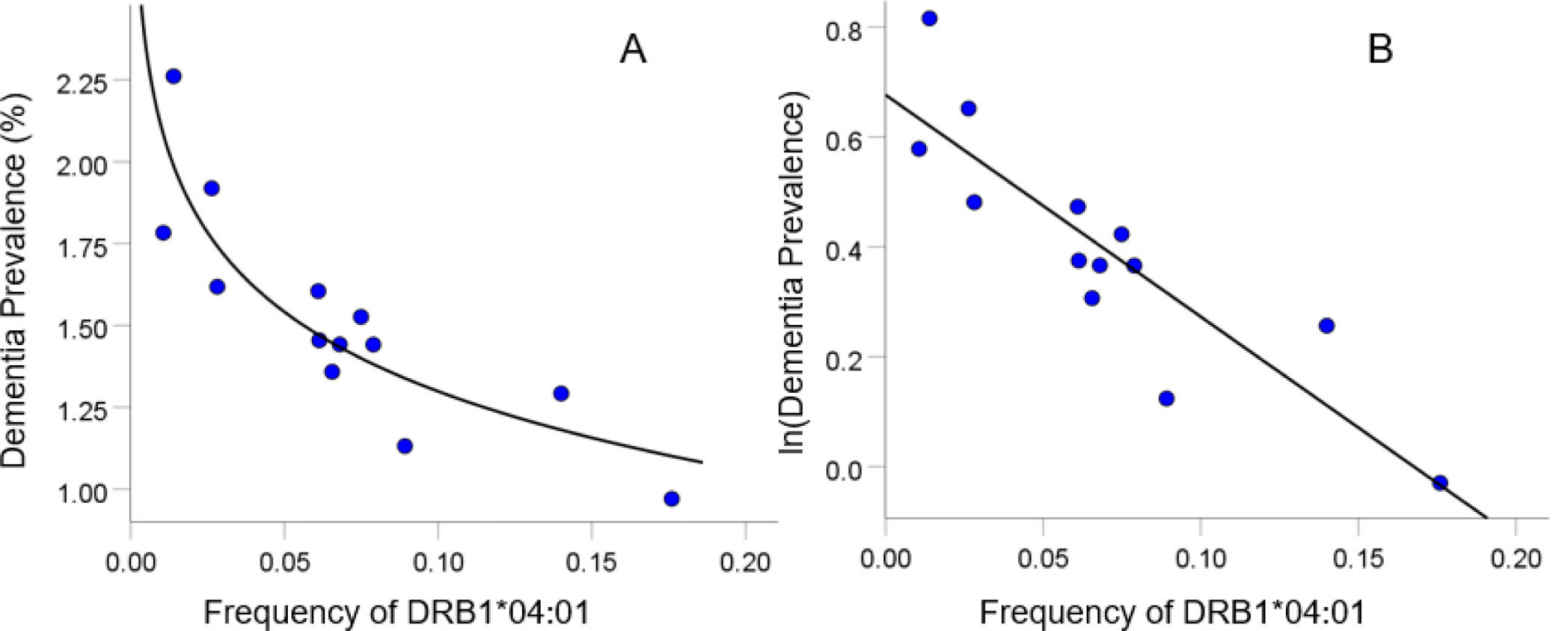
Example from a presumed protective HLA allele. Dementia prevalence for 13 CWE countries (for which DRB1*04:01 frequency was available) is plotted against the corresponding DRB1*04:01 allele frequency in the original (percentage) dementia prevalence scale (A) and its natural log transformed values (B). The fitted line is an exponential function (A) that becomes a linear function in the log-transformed prevalence scale (B). The statistics for the linear case are: Pearson correlation *r* = −0.877, *P* < 0.0001; $r^{'}=\text{atanh}(r)$ = −1.363.

**Figure 2.**
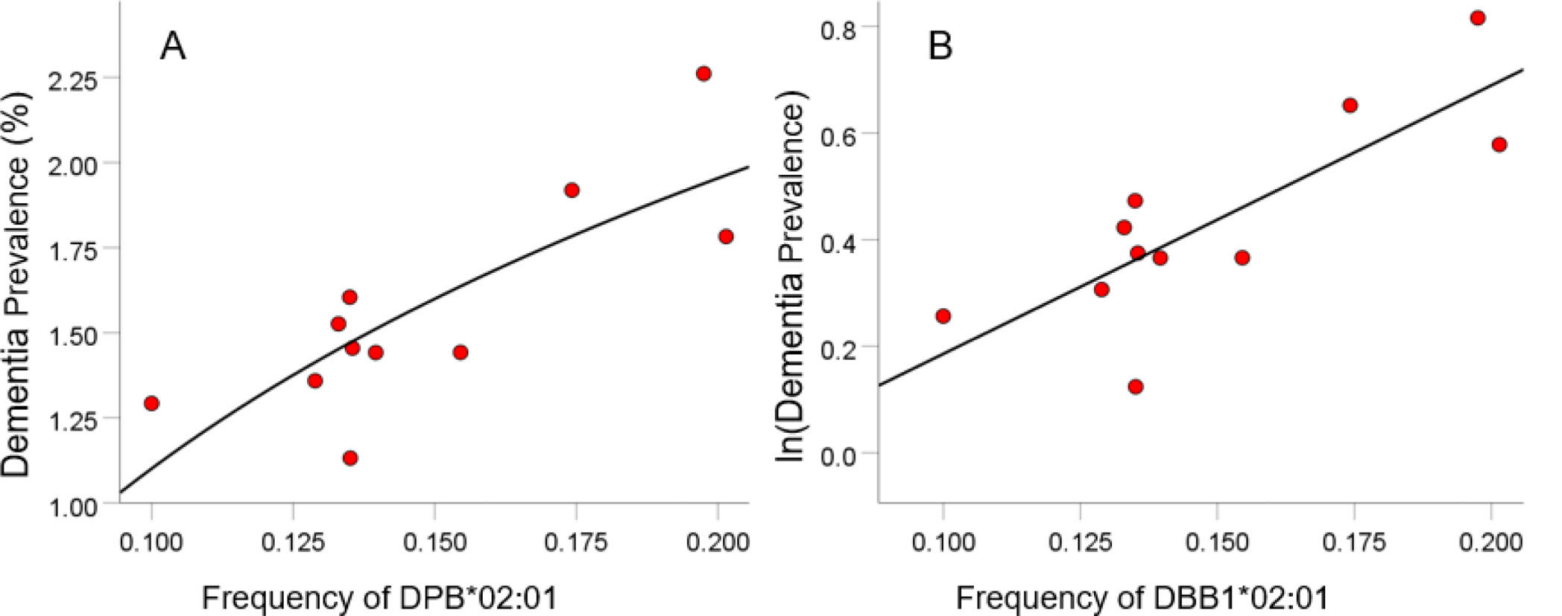
Example from a presumed susceptibility HLA allele. Dementia prevalence for 11 CWE countries (for which DPB1*02:01 frequency was available) is plotted against the corresponding DPB*02:01 allele frequency in the original (percentage) dementia prevalence scale (A) and its natural log transformed values (B). The fitted line is an exponential function (A) that becomes a linear function in the log-transformed prevalence scale (B). The statistics for the linear case are: Pearson correlation *r* = −0.805, *P* = 0.003; $r^{'}=\text{atanh}(r)$ = 1.112.

HLA-disease profiles consist of correlations between allele frequency and disease prevalence, suitably Fisher z-transformed (Equation 1) to normalize their distribution for further analyses. We showed previously^1^ that dementia prevalence varies in an exponential fashion with allele frequency, such that the logarithm of disease prevalence is a linear function of allele frequency. Therefore, the correlation entered in a HLA-disease profile is that computed using the natural log-transformed disease prevalence and allele frequency. Two examples are illustrated in Figures 1 and 2, one for a presumed dementia protective allele (DRB1*04:01; Figure 1) and another for a presumed dementia susceptibility allele (DPB1*02:01; Figure 2).

### HLA profiles of Parkinson’s disease and dementia

**Figure 3.**
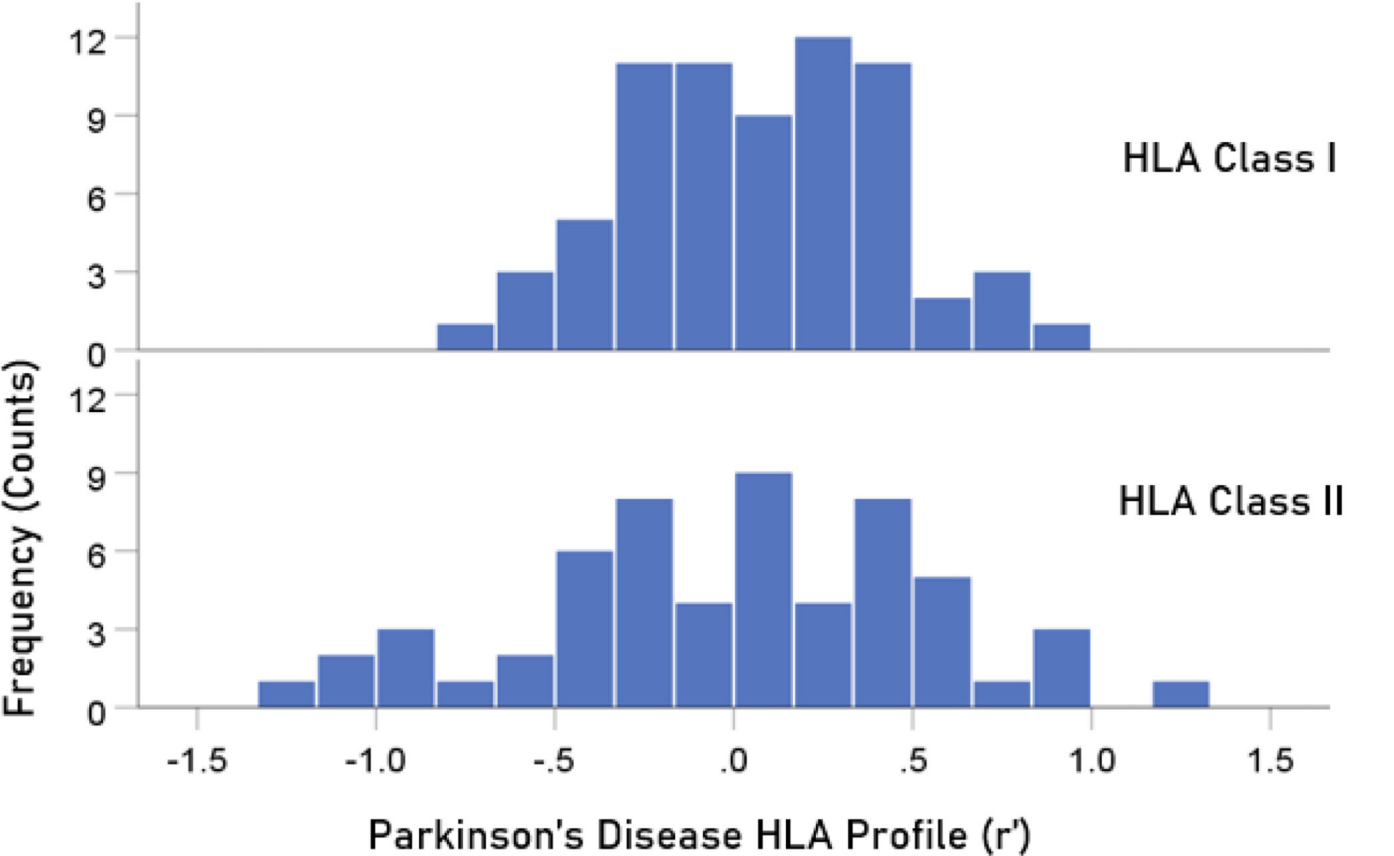
Frequency distribution of HLA profiles for Parkinson’s disease. N = 68 alleles for Class I and 59 alleles for Class II.

**Figure 4.**
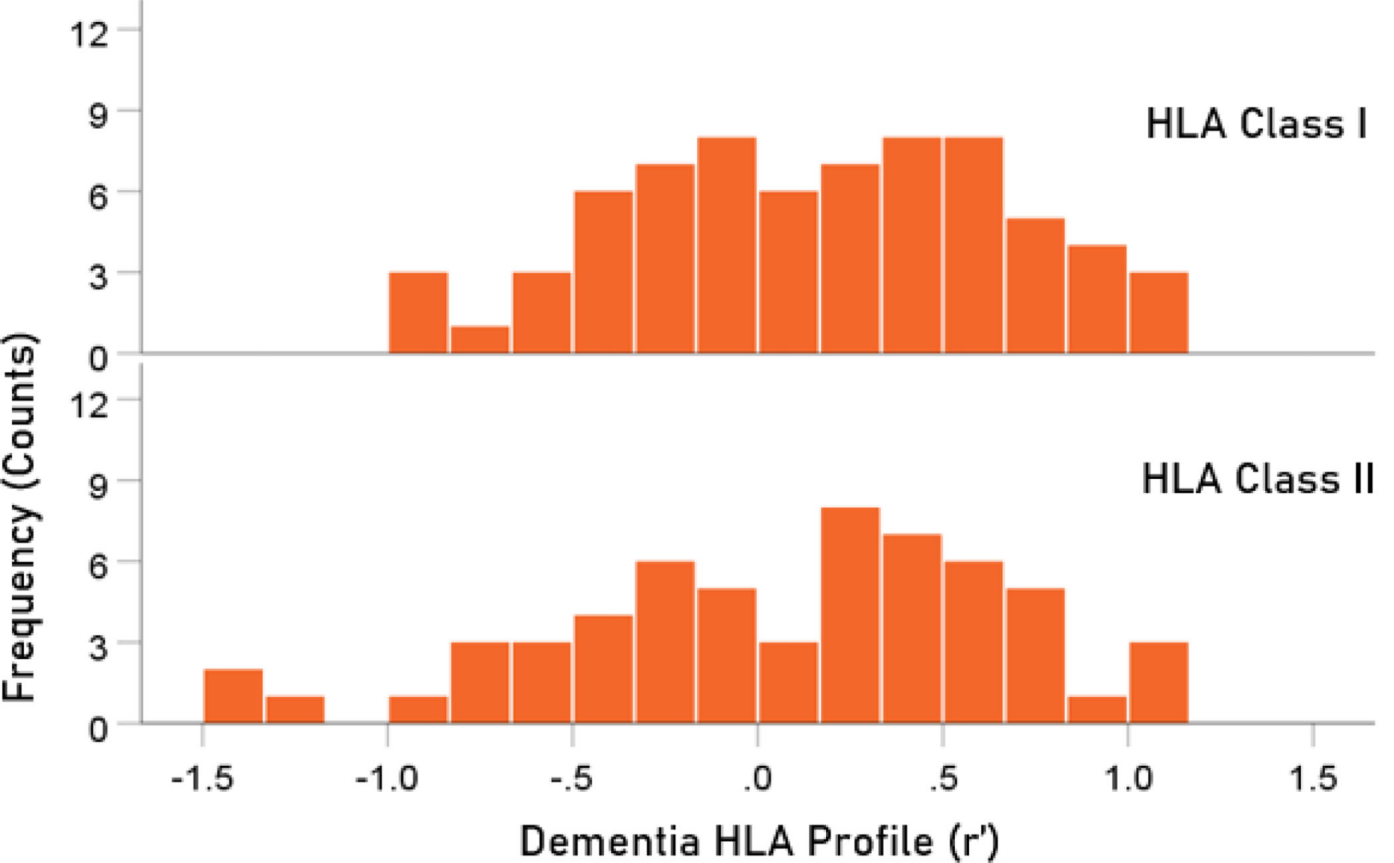
Frequency distribution of HLA profiles for dementia. N = 68 alleles for Class I and 59 alleles for Class II.

**Figure 5.**
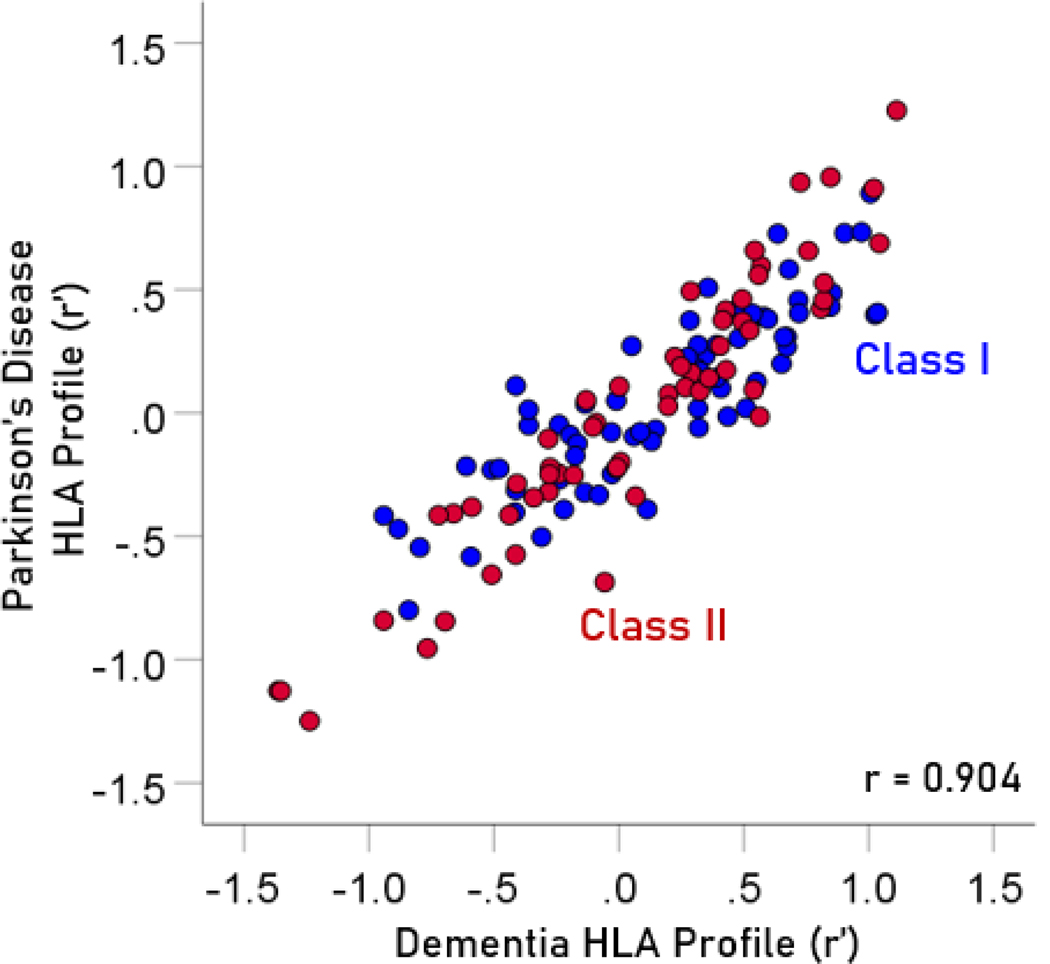
The HLA profile ($r^{'}$) of Parkinson’s disease is plotted against the HLA profile of dementia. The two HLA disease profiles were highly correlated. N = 127.

**Figure 6.**
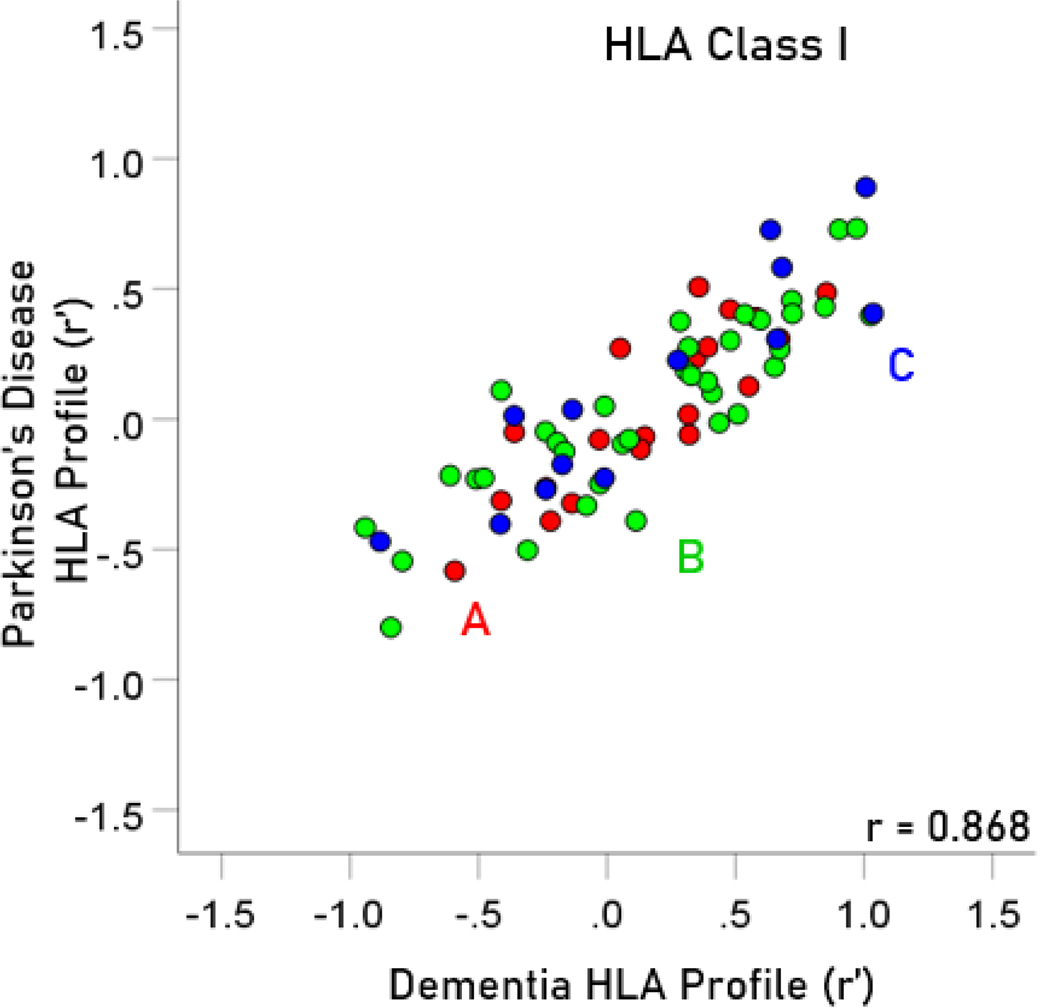
The HLA Class I profile of Parkinson’s disease is plotted against the HLA Class I profile of dementia. The two HLA disease profiles were highly correlated. N = 68 alleles.

**Figure 7.**
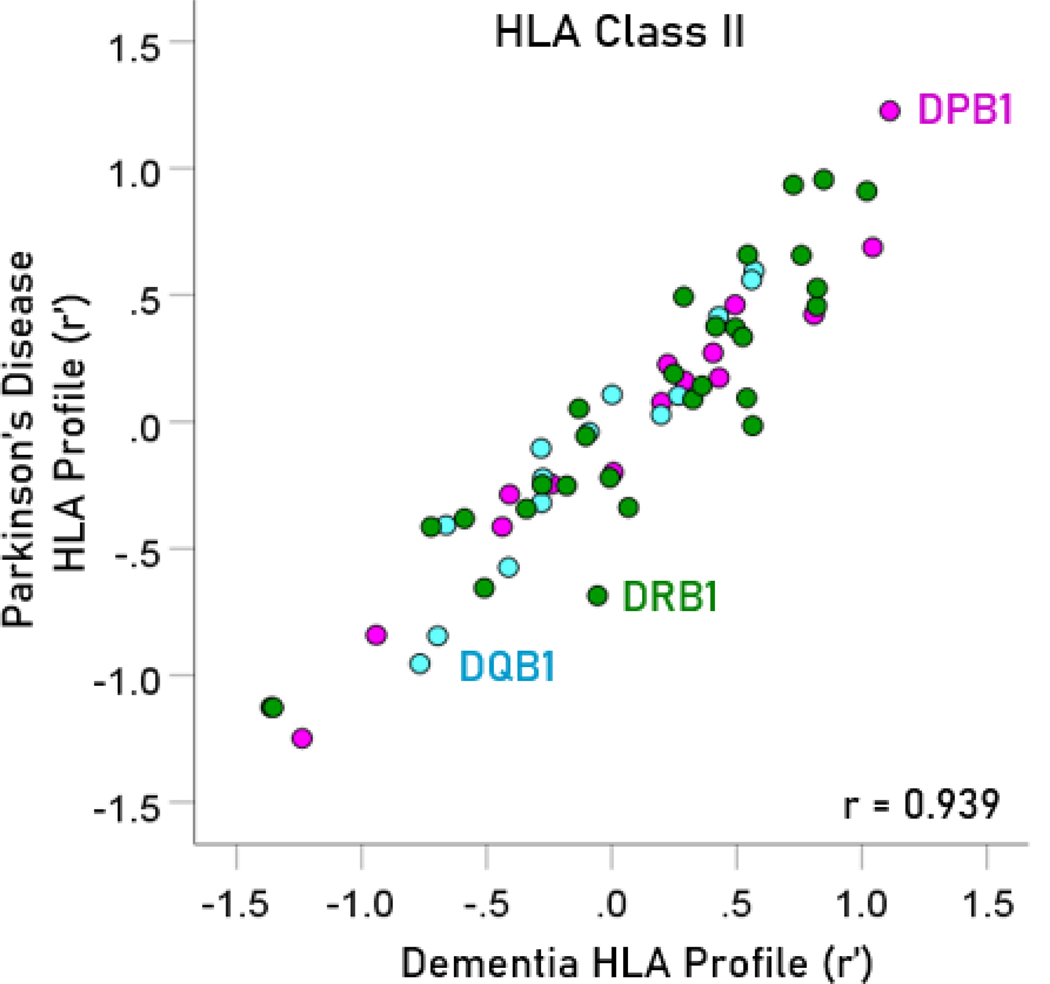
The HLA Class II profile of Parkinson’s disease is plotted against the HLA Class II profile of dementia. The two HLA disease profiles were highly correlated. N = 59 alleles.

The frequency distributions of alleles in HLA profiles for Parkinson’s disease and dementia (Table 2) are shown in Figures 3 and 4, respectively. It can be seen that they are similar, with a broad overlap. The HLA profiles of the two diseases were positively and highly significantly correlated (Figure 5) (r = 0.904, P = 6.4 × 10^−48^, N = 127). This positive association extended across the two HLA classes (color coded in Figure 5). More specifically, the correlation between the two disease HLA profiles was r = 0.868 (P = 4.8 × 10−22, N = 69) for Class I (Figure 6) and r = 0.939 (P = 1.3 × 10−27, N = 58) for Class II (Figure 7). In addition, this positive correlation extended across the 3 genes of Class I and the 3 genes of Class II (color coded in Figures 6 and 7, respectively). More specifically, the correlations between disease profiles were as follows: r = 0.854 (Class I, gene A, P = 2.0 × 10−6, N = 20), 0.867 (Class I, gene B, P = 7.8 × 10−12, N = 30), 0.903 (Class I, gene C, P = 2.3 × 10−5, N = 13), 0.974 (Class II, gene DPB1, P = 9.5 × 10−10, N = 15), 0.957 (Class II, gene DQB1, P = 8.8 × 10−8, N = 14), 0.914 (Class II, gene DRB1, P = 4.1 × 10−12, N = 29).

### Analysis of counts of signed correlations

The two-way distribution of the counts of signed correlations between allele frequency and disease prevalence is given in Table 3. There was a highly significant positive association (chi-square = 73.89, P (2-sided) = 8.2 × 10−18; Fisher’s exact test (2-sided) P = 4.4 × 10−19; phi = 0.763).

This positive association was present in both HLA Class I (Table 4) and Class II (Table 5). For Class I: chi-square = 31.68, P (2-sided) = 1.8 × 10−8; Fisher’s exact test (2-sided) P = 1.4 × 10−8; phi = 0.678). For Class II, this association was stronger: chi-square = 43.18, P (2-sided) = 5.0 × 10−11; Fisher’s exact test (2-sided) P = 5.2 × 10−12; phi = 0.863). Similar results were obtained for each one of the six alleles (Table 6).

### Comparison of Parkinson’s disease and dementia with respect to protective and susceptibility alleles

#### Protective alleles.

There were 48 alleles protective for both diseases. The magnitude of $r^{'}$ did not differ significantly between the two diseases (P = 0.221, paired t-test); for Parkinson’s disease, $r^{'}$ (mean ± SEM) was −0.403 ± 0.043, and for dementia −0.440 ± 0.050. Very similar results were obtained when the data were analyzed separately by a paired t-test for Class I and Class II alleles: for Class I, P = 0.156, N = 24, and for Class II, P = 0.824, N = 24).

#### Susceptibility alleles.

There were 64 susceptibility alleles for both diseases. The magnitude of $r^{'}$ was significantly higher for dementia than for Parkinson’s disease (P = 7.9 × 10−9, paired t-test); for Parkinson’s disease, $r^{'}$ (mean ± SEM) was 0.338 ± 0.032, and for dementia 0.552 ± 0.033. Very similar results were obtained when the data were analyzed separately by a paired t-test for Class I and Class II alleles: for Class I, P = 4.1 × 10−7, N = 34, and for Class II, P = 0.003, N = 30).

### Analyses with different sample sizes

For the analyses above, we used a minimum sample size of 9 countries as a reasonable choice of sample size. However, we also computed HLA profiles for all available samples sizes ≥ 3 and calculated correlations between these PD and dementia HLA profiles to check for consistency of their correlation. The results are shown in Table 7. It can be seen that a highly significant positive correlation between the 2 disease profiles was obtained for all cases of N ≥ 3.

## Discussion

**Figure 8.**
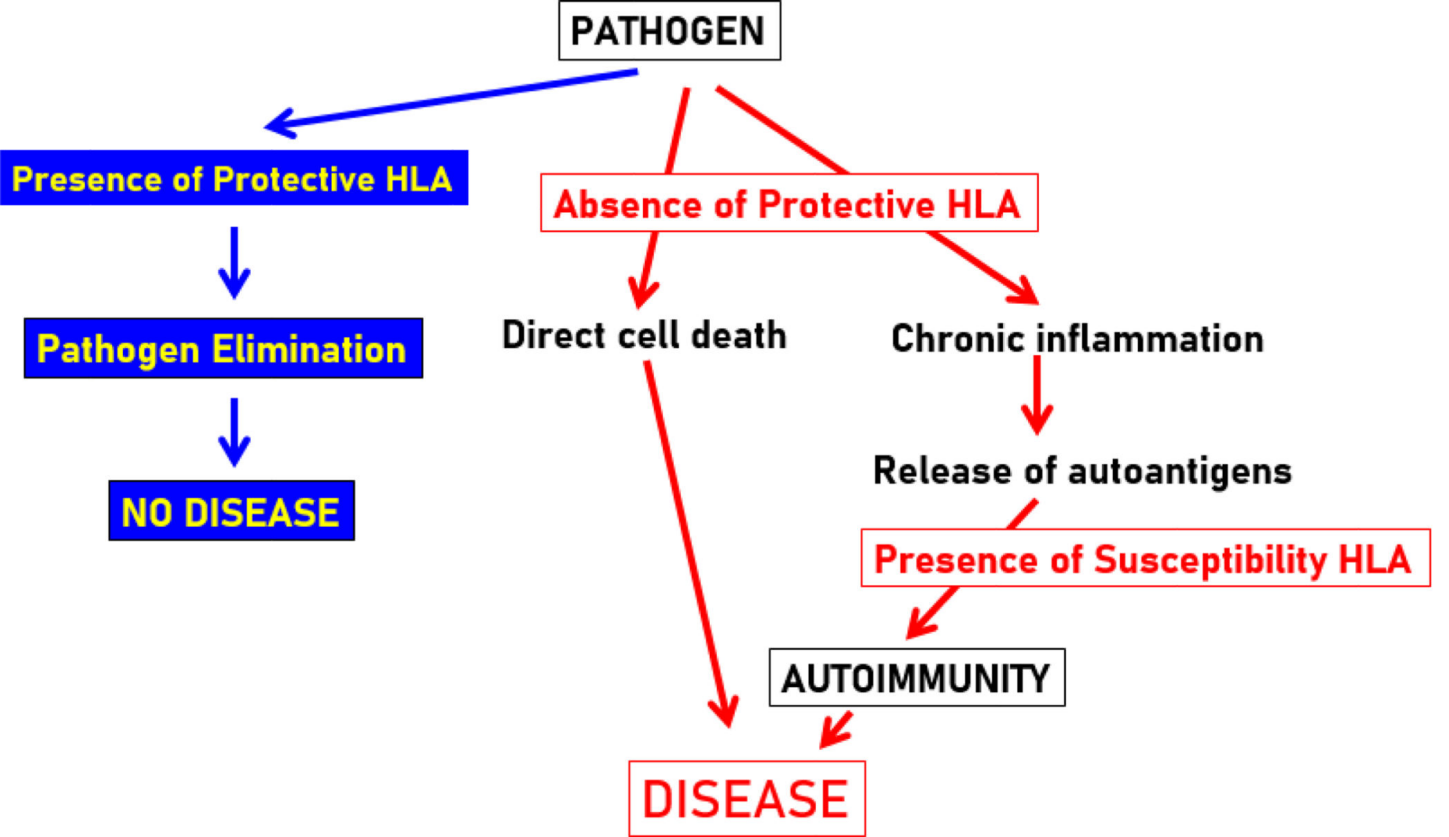
Diagram illustrating putative protective and susceptibility roles of HLA in disease outcomes.

Here we used an across-countries immunogenetic epidemiological approach^22^ to identify HLA profiles for dementia and Parkinson’s disease and evaluate their correspondence using data obtained from 14 countries in Continental Western Europe. The results demonstrated that the HLA profiles of the two diseases are remarkably similar when examined in aggregate and separately for each of the Class I and Class II alleles. However, when considered with regard to HLA protection or susceptibility, the correlation of susceptibility HLA alleles with dementia was significantly stronger than their correlation with Parkinson’s disease, whereas there was no significant difference regarding the correlation of protective/preventive alleles with dementia or Parkinson’s disease. These findings extend previous research demonstrating highly similar HLA DRB1 profiles in dementia and Parkinson’s disease^3^ to a large number of other Class I and Class II HLA alleles and point to increased immunogenetic susceptibility to dementia relative to Parkinson’s disease. Our view of how HLA could be involved in prevention/protection from, and susceptibility to, these and other diseases is discussed below and exemplified in the schematic diagram of Figure 8.

It is well-established that the HLA system evolved for, and is explicitly involved in, pathogen elimination. Thus, with respect to protective effects identified here, we assume that protective HLA alleles exert their effects via elimination of pathogens, thereby preventing deleterious downstream health effects. Our finding that protective HLA profiles (i.e., alleles with negative $r^{'}$) did not differ significantly between the two diseases is consistent with evidence implicating similar families of pathogens in both Parkinson’s disease and dementia^6^–^8^, ^27^–^34^.

In the absence of HLA protection against pathogens, disease may result from directly damaging effects of a pathogen on cells or as a result of susceptibility HLA alleles (i.e., alleles with positive $r^{'}$) that promote autoimmunity (i.e., production of autoantibodies) due to chronic inflammation^16^,^35^. This is in accord with evidence documenting inflammatory and autoimmune processes in both dementia and Parkinson’s disease^9^,^27^,^36^ as well as evidence of autoantibodies in both conditions^37^–^41^. Here, susceptibility (i.e., positively associated) alleles occurred more frequently than protective (i.e., negatively associated) alleles, indicating a preponderance of HLA alleles that may increase susceptibility to autoimmunity.

At the individual level, HLA composition plays a critical role in influencing health vs immune-mediated disease outcomes due to the protective role of HLA in eliminating foreign antigens^42^ and to its predisposing role in autoimmunity^16^. As previously noted, HLA is the most highly polymorphic region of the human genome. This is notable in that subtle alterations in HLA have been shown to affect the binding groove, resulting in differential binding affinity of antigens^43^,^44^. We have hypothesized that protection against diseases including dementia and Parkinson’s disease conferred by specific HLA alleles is related to their superior ability to bind, and therefore eliminate, harmful antigens. On the other hand, inability to bind antigens and mount an immune response is posited to result in persistent antigens that may directly damage cells and/or may potentially stimulate chronic inflammatory responses and/or autoimmunity^45^–^47^ and ultimately clinical disease^42^. Dementia commonly occurs in individuals with Parkinson’s disease^48^. The extent to which the co-occurrence may be a result of pathogen-driven cell death or autoimmunity is unclear.

It is worth noting that the persistent antigen hypothesis is complementary rather than inconsistent with prevailing theories implicating aggregated self-proteins in dementia and Parkinson’s disease. Indeed, both amyloid-β and alphasynuclein, proteins associated with dementia and Parkinson’s disease, respectively, have been shown to exhibit anti-microbial properties^29^,^49^,^50^. That is, growing research suggests that aggregated proteins that have long been characterized as the hallmark pathological characteristics of neurodegenerative disorders may initially reflect a protective immune response to infections^29^,^49^,^50^. If, however, foreign antigens persist due to lack of HLA-antigen congruence as suggested by the persistent antigen hypothesis^42^, the amyloid-β and/or alphasynuclein protein aggregation may continue, resulting in unchecked protein deposition. In turn, aggregated self-proteins may be recognized as foreign antigens, stimulating further immune system reactivity^47^.

Here we found no differences between dementia and Parkinson’s disease prevalence with respect to protective HLA alleles for both conditions; however, the association of susceptibility HLA alleles was stronger with dementia than the association of those same alleles with Parkinson’s disease. These findings suggest that similar immunogenetic mechanisms along the lines of those discussed above (i.e., elimination of pathogens) are involved in protection against these conditions but that additional factors are involved in moderating HLA-mediated autoimmunity in dementia and Parkinson’s disease, including variations in apoE^51^,^52^, modifiable risk factors including diet, exercise, and smoking, among others^4^,^5^,^53^, and other environmental exposures that have been differentially associated with dementia and Parkinson’s disease^4^,^5^. These issues represent important areas for future investigation.

A broad link between HLA and neurodegenerative diseases such as dementia and Parkinson’s disease has been increasingly recognized^1^–^3^,^9^,^17^–^21^; however, the influence of specific genes on these diseases has not been clearly established and findings have often been inconsistent. For example, we have identified population-level protective effects of DRB1*15:01 on dementia and Parkinson’s disease here, and have previously shown that DRB1*15:01 binds with viruses linked to dementia and Parkinson’s disease with very high affinity^54^. In contrast, another recent study identified DRB1*15:01 as a risk factor for Alzheimer’s dementia^19^; however, in that study HLA was imputed rather than directly sequenced, and the findings regarding DRB1*15:01 were limited to men lacking the ApoE4 risk gene. Thus, additional research using direct sequencing of HLA is warranted to further evaluate the association of HLA alleles, including DRB1*15:01, to dementia and to evaluate the effects of gender and other moderating factors. Similarly, although the findings regarding associations between HLA (including DRB1*15:01) and Parkinson’s disease are more compelling^55^–^57^, additional research is warranted to more conclusively establish HLA associations with Parkinson’s disease and to evaluate variations in HLA-disease associations across different populations given global variations in HLA. Determining specific HLA influences on diseases move beyond the common influence of inflammation in dementia and Parkinson’s disease to facilitate *in silico* analyses that permit identification of pathogen families that may contribute to inflammation and disease (e.g., ref^54^).

## Limitations and qualifications

There are several limitations/qualifications of this study, as follows. First, these results are based on correlations between disease prevalences and HLA allele frequencies in large populations of 14 CWE countries and, as such, they need to be validated in studies using assessments of disease and allele presence in specific individuals. Second, it is known that HLA-disease associations may vary from place to place^58^,^59^, and across countries^22^,^59^ and, therefore, the results of this study are properly applicable to the 14 CWE countries used here but could be extended to other countries/regions with further analyses, as done for malaria^22^. Finally, it should be noted that the dementia population(s) in the GBD study used here^4^ include dementia cases from Parkinson’s disease. However, the estimated percentage of dementia attributable to Parkinson’s disease in the population is only 3–4% globally^60^, and reducing the GBD dementia population counts accordingly would yield the same correlations between the adjusted dementia prevalence and HLA allele frequency, since the percentage above is generally consistent from country to country.

## Summary and Conclusions

The present study documents highly overlapping HLA profiles for dementia and Parkinson’s disease at the population level in Continental Western Europe, suggesting that similar population-level immunogenetic mechanisms contribute to prevention and/or susceptibility to both conditions. With respect to prevention, HLA plays a major role in the elimination of foreign antigens and, hence, its protective role found here can be attributed to the elimination of potential pathogens implicated as putative causative agents for these diseases (e.g., human herpes viruses^31^). HLA also plays a major role in autoimmunity which may occur in the absence of protection and in the presence of ensuing chronic inflammation. In both protection (pathogen elimination) and susceptibility (autoimmunity) cases, the HLA overlap between dementia and Parkinson’s disease indicates a similarity/overlap in the family of putative pathogens and autoantigens, respectively. The identification of such pathogens and autoantigens could be further investigated (e.g., in silico) using information from the specific alleles involved in protection and susceptibility. Finally, the present findings and future prospects regarding identification of disease-associated pathogens and autoantigens based on a disease’s HLA-profile extend beyond common neurodegenerative diseases to include a wide range of conditions for which pathogens and/or autoimmunity have been implicated, both in specific regions and globally.

## Figures and Tables

**Table 1:** Distribution of 127 HLA alleles analyzed to Class and Genes.

	Class I (N = 69)			Class II (N = 58)		
Gene	A	B	C	DPB1	DQB1	DRB1
Count	20	36	13	15	14	29

**Table 2:** The signed z-transformed correlation coefficient ($r^{'}$) between 127 HLA alleles and disease prevalence.

	Allele	Class	N	$r^{'}$(*PD*)	$r^{'}$(*DEM*)
1	A*01:01	1	11	−0.263	−0.237
2	A*02:01	1	11	−0.583	−0.593
3	A*02:05	1	9	0.486	0.853
4	A*03:01	1	11	−0.313	−0.412
5	A*11:01	1	11	0.233	0.347
6	A*23:01	1	11	0.018	0.317
7	A*24:02	1	11	0.272	0.051
8	A*25:01	1	12	−0.078	−0.031
9	A*26:01	1	11	0.421	0.478
10	A*29:01	1	11	−0.067	0.147
11	A*29:02	1	11	−0.115	0.131
12	A*30:01	1	11	0.508	0.357
13	A*30:02	1	12	0.390	0.580
14	A*31:01	1	9	−0.051	−0.361
15	A*32:01	1	12	0.277	0.392
16	A*33:01	1	10	0.308	0.672
17	A*33:03	1	9	−0.059	0.320
18	A*36:01	1	10	0.126	0.551
19	A*68:01	1	11	−0.391	−0.220
20	A*68:02	1	10	−0.323	−0.137
21	B*07:02	1	10	−0.416	−0.941
22	B*08:01	1	12	−0.545	−0.797
23	B*13:02	1	11	−0.047	−0.240
24	B*14:01	1	11	0.100	0.409
25	B*14:02	1	10	0.457	0.719
26	B*15:01	1	10	−0.216	−0.611
27	B*15:17	1	9	0.728	0.903
28	B*15:18	1	9	0.302	0.479
29	B*18:01	1	12	0.381	0.597
30	B*27:02	1	10	−0.095	0.060
31	B*27:05	1	12	−0.090	−0.195
32	B*35:01	1	11	0.186	0.306
33	B*35:02	1	9	0.200	0.651
34	B*35:03	1	9	0.268	0.672
35	B*35:08	1	9	0.733	0.971
36	B*37:01	1	10	0.110	−0.412
37	B*38:01	1	9	0.398	1.026
38	B*39:01	1	11	0.375	0.284
39	B*39:06	1	9	−0.332	−0.080
40	B*40:01	1	12	−0.230	−0.509
41	B*40:02	1	12	0.050	−0.010
42	B*41:01	1	11	0.144	0.391
43	B*41:02	1	10	−0.014	0.436
44	B*44:02	1	12	−0.799	−0.841
45	B*44:03	1	12	−0.077	0.086
46	B*44:05	1	9	0.167	0.327
47	B*45:01	1	10	−0.248	−0.028
48	B*47:01	1	11	−0.391	0.113
49	B*49:01	1	11	0.431	0.847
50	B*50:01	1	10	0.019	0.509
51	B*51:01	1	10	0.402	0.535
52	B*52:01	1	10	0.276	0.317
53	B*55:01	1	11	−0.225	−0.479
54	B*56:01	1	9	−0.503	−0.309
55	B*57:01	1	12	−0.124	−0.166
56	B*58:01	1	9	0.405	0.721
57	C*01:02	1	9	−0.225	−0.011
58	C*03:03	1	9	−0.403	−0.416
59	C*04:01	1	9	0.406	1.035
60	C*05:01	1	9	−0.174	−0.174
61	C*06:02	1	9	0.037	−0.135
62	C*07:01	1	9	0.013	−0.362
63	C*07:02	1	9	−0.470	−0.883
64	C*07:04	1	9	−0.270	−0.238
65	C*12:02	1	9	0.727	0.636
66	C*12:03	1	9	0.890	1.007
67	C*14:02	1	9	0.582	0.681
68	C*15:02	1	9	0.307	0.660
69	C*16:01	1	9	0.226	0.275
70	DPB1*01:01	2	11	−0.841	−0.941
71	DPB1*02:01	2	11	1.226	1.112
72	DPB1*02:02	2	10	0.272	0.405
73	DPB1*03:01	2	11	0.462	0.493
74	DPB1*04:01	2	11	-1.248	-1.238
75	DPB1*04:02	2	11	−0.414	−0.438
76	DPB1*05:01	2	11	−0.287	−0.408
77	DPB1*06:01	2	10	0.077	0.199
78	DPB1*09:01	2	9	0.688	1.043
79	DPB1*10:01	2	10	0.423	0.809
80	DPB1*11:01	2	9	−0.199	0.007
81	DPB1*13:01	2	10	0.161	0.291
82	DPB1*14:01	2	11	0.227	0.223
83	DPB1*17:01	2	9	0.174	0.429
84	DPB1*19:01	2	11	−0.246	−0.241
85	DQB1*02:01	2	12	−0.574	−0.413
86	DQB1*02:02	2	11	0.028	0.197
87	DQB1*03:01	2	13	0.595	0.569
88	DQB1*03:02	2	13	−0.320	−0.280
89	DQB1*03:03	2	13	−0.105	−0.282
90	DQB1*04:02	2	13	−0.041	−0.091
91	DQB1*05:01	2	13	−0.222	−0.275
92	DQB1*05:02	2	10	0.560	0.560
93	DQB1*05:03	2	12	0.416	0.428
94	DQB1*06:01	2	11	0.104	0.267
95	DQB1*06:02	2	14	−0.844	−0.696
96	DQB1*06:03	2	13	−0.954	−0.767
97	DQB1*06:04	2	12	−0.407	−0.662
98	DQB1*06:09	2	9	0.107	0.001
99	DRB1*01:01	2	14	−0.414	−0.722
100	DRB1*01:02	2	11	0.094	0.540
101	DRB1*01:03	2	11	−0.338	0.067
102	DRB1*03:01	2	13	0.370	0.494
103	DRB1*04:01	2	13	-1.126	-1.363
104	DRB1*04:02	2	11	0.657	0.758
105	DRB1*04:03	2	12	0.335	0.524
106	DRB1*04:04	2	13	−0.248	−0.278
107	DRB1*04:05	2	9	0.910	1.020
108	DRB1*04:07	2	12	0.189	0.248
109	DRB1*04:08	2	9	−0.343	−0.341
110	DRB1*07:01	2	12	−0.221	−0.007
111	DRB1*08:01	2	13	−0.056	−0.104
112	DRB1*08:03	2	11	−0.686	−0.057
113	DRB1*09:01	2	12	0.052	−0.131
114	DRB1*10:01	2	14	0.493	0.288
115	DRB1*11:01	2	14	0.956	0.847
116	DRB1*11:02	2	12	−0.015	0.564
117	DRB1*11:03	2	12	0.456	0.820
118	DRB1*11:04	2	12	0.934	0.726
119	DRB1*12:01	2	13	−0.252	−0.180
120	DRB1*13:01	2	14	−0.656	−0.509
121	DRB1*13:02	2	14	−0.382	−0.589
122	DRB1*13:03	2	10	0.527	0.821
123	DRB1*13:05	2	10	0.088	0.324
124	DRB1*14:01	2	14	0.142	0.361
125	DRB1*15:01	2	13	-1.127	-1.353
126	DRB1*15:02	2	10	0.376	0.416
127	DRB1*16:01	2	10	0.659	0.544

**Table 3:** Two-way table of the distribution of signed correlation counts in the whole sample (N = 127 alleles).

		Dementia		
		Negative	Positive	Total
Parkinson’s disease	Negative	48	10	58
	Positive	5	64	69
	Total	53	74	127

**Table 4:** Two-way table of the distribution of signed correlation counts in HLA Class I (N = 69 alleles).

		Dementia		
		Negative	Positive	Total
Parkinson’s disease	Negative	24	7	31
	Positive	4	34	38
	Total	28	41	69

**Table 5:** Two-way table of the distribution of signed correlation counts in HLA Class II (N = 58 alleles).

		Dementia		
		Negative	Positive	Total
Parkinson’s disease	Negative	24	3	27
	Positive	1	30	31
	Total	25	33	58

**Table 6:** Two-way tables of the distribution of signed correlation counts in all six HLA Class I and II alleles. Values in parentheses are two-sided probability values of the Fisher’s exact test.

				Dementia		
				Negative	Positive	Total
Parkinson’s disease	Class I	A (P = 0.003)	Negative	7	3	10
			Positive	0	10	10
			Total	7	13	20
		B (P = 0.0001)	Negative	12	4	16
			Positive	2	18	20
			Total	14	22	36
		C (P = 0.021)	Negative	5	0	5
			Positive	2	6	8
			Total	5	6	13
	Class II	DPB1 (P = 0.002)	Negative	5	1	6
			Positive	0	9	9
			Total	5	10	15
		DQB1 (P = 0.0003)	Negative	8	0	8
			Positive	0	6	6
			Total	8	6	14
		DRB1 (P = 0.00002)	Negative	11	2	13
			Positive	1	15	16
			Total	12	17	29

**Table 7:** Correlation coefficients between $r^{'}$(***PD***) and $r^{'}$(***DEM***) at various levels of numbers of alleles available in the 14 CWE countries.

Number of countries with N alleles	Number of distinct alleles	Correlation between $r^{'}$(*PD*) and $r^{'}$(*DEM*)	P –value
≥ 3	266	0.748	P < 0.0001
≥ 4	217	0.798	P < 0.0001
≥ 5	186	0.852	P < 0.0001
≥ 6	171	0.877	P < 0.0001
≥ 7	162	0.878	P < 0.0001
≥ 8	151	0.884	P < 0.0001
≥ 9	127	0.904	P < 0.0001
≥ 10	95	0.903	P < 0.0001
≥ 11	73	0.920	P < 0.0001
≥ 12	40	0.941	P < 0.0001
≥ 13	19	0.964	P < 0.0001
14	7	0.946	P = 0.0012

## Data Availability

The datasets analyzed for this study are publicly available. The data can be found in the Allele Frequency Net Database (allelefrequencies.net) and in publications^4^,^5^,^23^.

## Abbreviations

HLA: Human Leukocyte Antigen
